# Gridded sea surface hydrographic data along two Salish Sea passenger ferry routes, 2001–2025

**DOI:** 10.1016/j.dib.2026.113279

**Published:** 2026-09-21

**Authors:** Ian T. Black, Alice O.V. Bui, Bennit L. Mueller, Kohen W. Bauer

**Affiliations:** a Oregon State University, College of Earth, Ocean, and Atmospheric Sciences, Corvallis, OR, USA; b University of Victoria, Ocean Networks Canada, Victoria, BC, Canada; c Carbon to Sea Initiative, Halifax, NS, Canada

**Keywords:** Oceanography, Underway, Flowthrough, Temperature, Salinity, Density, Oxygen

## Abstract

Since 2001, several passenger ferries connecting Vancouver Island and mainland British Columbia, Canada have collected sea surface hydrographic data within the Salish Sea. Originally part of two separate monitoring projects in the early 2000s and now maintained by Ocean Networks Canada (ONC), archived and real-time data from these vessels are open to the public. To date, several vessels with scientific underway systems have collected data between Tsawwassen-Nanaimo (TWS-NNO, ∼70 km) and Tsawwassen-Swartz Bay (TWS-SWB, ∼44 km), sometimes up to 8 times per day. This data article describes how the original time-series were obtained, the performance of additional quality assessment, and the steps for creating curated datasets consisting of spatially binned (0.01°) transits of surface ocean water properties in the Salish Sea between 2001–2025. During this period, 30,000+ transits along both routes collected sea surface temperature, practical salinity, and dissolved oxygen; providing dense coverage for a variety of inter-seasonal and inter-annual analyses. Pathways for uncertainty estimation and validation are discussed. Python 3 example code is provided for compiling and processing new data collected after 2025 to enable users to extend this dataset for future analyses.

Specifications Table

**Table:** 

Subject	Earth & Environmental Sciences
Specific subject area	Oceanography
Type of data	Processed, Binned
Data collection	Data were acquired from three passenger ferries with automated underway systems. The intake position is located nominally at 3 m depth and 20 m forward of amidships. TWS-NNO indicates the ferry route between Tsawwassen to Nanaimo (Duke Point). TWS-SWB indicates the ferry route between Tsawwassen to Swartz Bay. Navigation (NAV) data were collected by: -Thrane and Thrane Sailor GPS -Hemisphere GNSS MV104S GPS Compass -Hemisphere GNSS V200S GPS Compass Thermosalinograph (TSG) data were collected by: -Sea-Bird Electronics SBE21 Thermosalinograph -Sea-Bird Electronics Seakeeper -Sea-Bird Electronics SBE45 Microthermosalinograph Dissolved oxygen (OXYSENSOR) data were collected by: -Aanderraa Oxygen Optode 3835 Intake temperature (TEMPSENSOR) data were collected by (TWS-NNO only): -TURO PT-100 RTD -Sea-Bird Electronics SBE38 Digital Thermometer Data are binned into 0.01° grid cells (∼720 m N-S x 1100 m E-W) with timestamps indicating the approximate time the ferry departed a terminal. 31,510 transits have been compiled for TWS-NNO. 33,648 transits have been compiled for TWS-SWB.
Data source location	Location: Salish Sea, British Columbia, Canada (48.67 to 49.26 °N, −123.98 to −122.98 °E) Time Period: TWS-NNO -> January 2003 – November 2025 TWS-SWB -> December 2001 – December 2025
Data accessibility	Repository name: Zenodo Data Identification Number (Manuscript Version 1.2.1): 10.5281/zenodo.22116327 Direct URL (Manuscript Version 1.2.1): https://doi.org/10.5281/zenodo.22116327 Raw and initial quality assessed data are open and available through the Ocean Networks Canada Data Portal (https://data.oceannetworks.ca). A complete list of the original data DOIs may be found in the References section and within the Zenodo repository. An example Python notebook is provided to enable users to extend this dataset as new data become available along each route.
Related research article	None

## 1 Value of the Data

• More than two decades of observations captured along two passenger ferry routes in the Salish Sea make up this curated dataset. Despite gaps in collection, with a median of 8 transits per day, these data may help support high-resolution spatiotemporal analyses along each route.
• Early original data (2005, 2012–2013) have been used in studies focused on the Fraser River [1,2]. This new curated dataset may be used to support newer studies involving the Fraser River, one of the most important freshwater inputs to the Strait of Georgia.
• These curated data may be used for rapid comparison with modeled and regional gridded satellite data products encompassing the Strait of Georgia.
• Gridded transits may help support physical, biogeochemical, and ecological studies of the Salish Sea when paired with data from other academic institutions, the Canada Department of Fisheries and Oceans Canada, and other nearby Ocean Networks Canada assets, such as the Strait of Georgia subsurface cabled nodes or high frequency radar.
• These curated data may be useful for teaching oceanographic and data analysis principles in the classroom.

## 2 Background

Since 2001, several vessels operated by British Columbia Ferries (BCF) have become platforms of opportunity that facilitate scientific sampling along frequently transited transects within the Salish Sea. Between 2001–2011, the Canada Department of Fisheries and Oceans (DFO) installed several oceanographic instruments aboard the MV Spirit of Vancouver Island (SoVI) to facilitate scientific sampling along its primary route between Tsawwassen and Swartz Bay (TWS-SWB) (Fig. 1). As a separate project in 2003–2006, the Strait of Georgia Ecosystem Project (STRATOGEM, https://www.stratogem.ubc.ca/) installed several instruments aboard the MV Queen of New Westminster (QNW), which primarily operated along the Tsawwassen and Nanaimo (TWS-NNO) route (Fig. 1). Toward the end of the STRATOGEM Project, instruments were removed from the QNW and installed aboard the MV Queen of Alberni (QoA). In 2012, motivated to maintain data continuity, Ocean Networks Canada (ONC) and BCF entered cooperative agreement and instruments were reinstalled on the SoVI and QoA with an emphasis on providing open data to the public. Since then, several other passenger vessels have become host to scientific underway systems along different routes within the Salish Sea, with data made available by ONC in real-time and as archived time-series via the ONC Oceans Data Portal (https://data.oceannetworks.ca) [3]. This data article focuses solely on the curation of the original temperature, salinity, and oxygen data collected along the two longest operating routes, TWS-NNO and TWS-SWB.

**Fig. 1:**
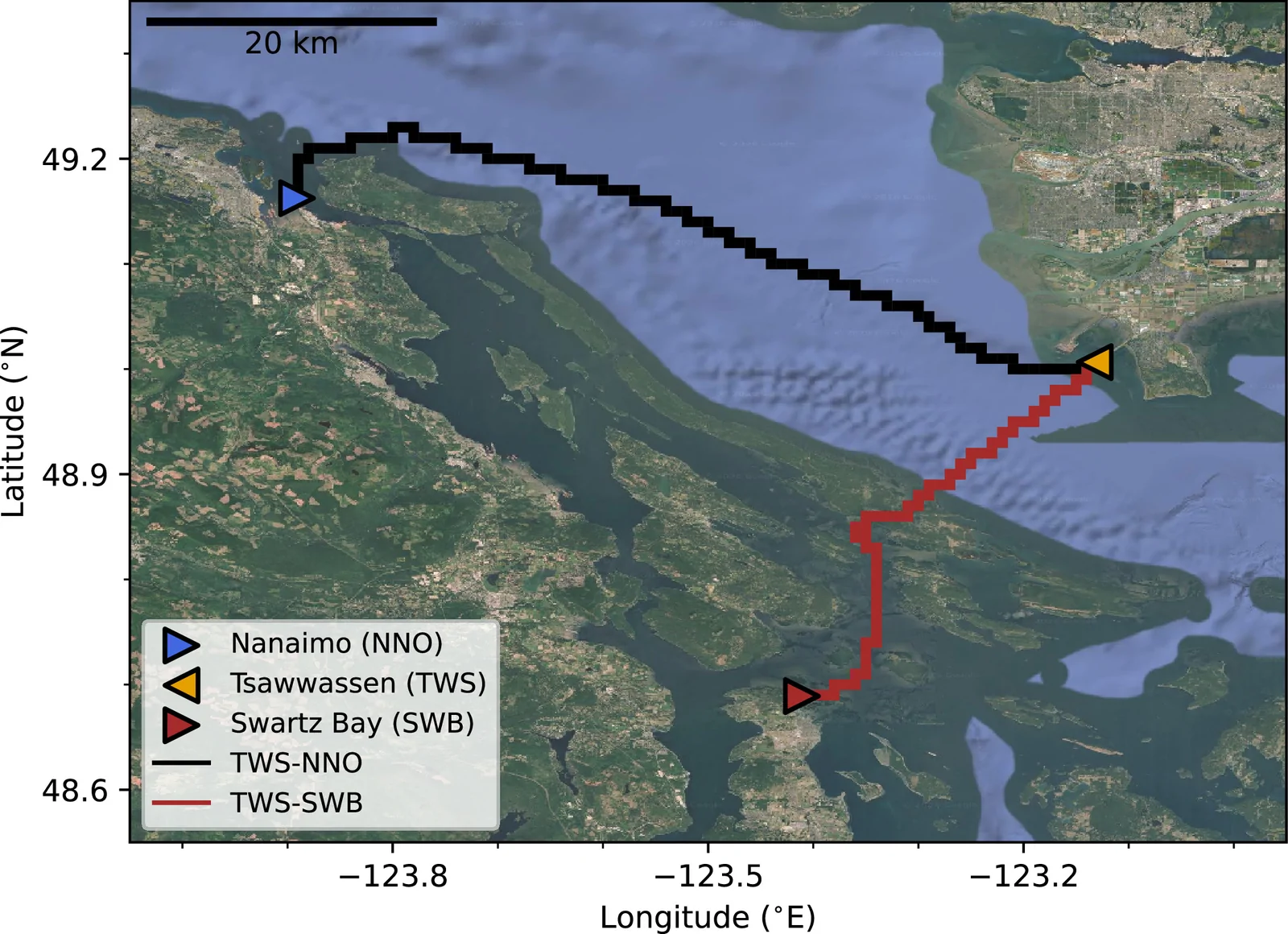
Map of primary ferry routes represented as 0.01° grid cells for the TWS-NNO (black) and TWS-SWB (brown) routes. Navigation data are from a transit from each ferry that occurred on 2025–07–04. Route paths may vary several kilometers across the dataset. Within the ONC Oceans Data Portal these routes have the location Code identifiers of TWDP (TWS-NNO) and TWSB (TWS-SWB). Imagery ©2026 NASA, Map Data ©2026 Google.

The data distributed by ONC can quickly become complex with a variety of quality flags and secondary engineering variables to parse through. A few obscure system issues have also created gaps or data that have similar appearance to real oceanographic features and may not always be captured in annotations or automated quality assessment results, requiring careful review. Data arriving from the ONC Oceans Data Portal is also distributed as a 1-dimensional time-series with no unique identifier for transits or end members along a route, requiring users to reformat data to perform spatial analyses.

Previous studies have used a subset and binned version of the original data for analysis. Temperature, salinity, and chlorophyll data from 2005 along the TWS-NNO route have been used by Halverson and Pawlowicz [1] to study the biophysical impacts of the Fraser River Plume. Data along the same route from 2012 to 2013 were then used again by Halverson and Pawlowicz [2] to support analysis of surface ocean current forcings in the region. The original time-series along the TWS-NNO route has been used to evaluate the SalishSeaCast NEMO Model (https://salishsea.eos.ubc.ca/nemo/). In this work, we further curate ferry data in a way that helps support similar applications with more modern data and facilitates rapid comparison along each route through space and time.

## 3 Data Description

A Network Common Data Form (netCDF, .nc) file is provided for each route and each file is made up of two groups (Table 1). The root group contains the gridded science data represented as the grid cell mean value and is accessible on file read without specifying a hierarchical group name. The ‘statistics’ group contains statistical information and descriptive variables (detailed further in Table 1) that may help users further analyze and validate the data. File variables are compressed to level 6 using zlib and each file requires ∼700 mb of disk space. Uncompressed and eagerly loaded, the root group of a single file may take up to ∼2 gb of memory, while the statistics group may take ∼20 gb of memory. Metadata (e.g. Climate and Forecast (CF) standard names, valid ranges) are made available as attributes at the variable levels in each file. Files are named by route (*TWS-NNO_2003–2025_V1.2.1.nc and TWS-SWB_2001–2025_V1.2.1.nc)* and follow CF Conventions (CF-1.13).

**Table 1:** NetCDF file format and variable descriptions.

Root Group				
Category	Variable Name	Units	Description	Valid Min/Max or Values
dimension	time	minutes since 1970–01–01	The approximate time the ferry leaves the departure terminal.	Min: 2001–12–11T00:00:00Z
dimension	latitude	°N (decimal degrees)	The latitude of the grid cell. Represents the center of the cell.	Min: 48.67 Max: 49.26
dimension	longitude	°E (decimal degrees)	The longitude of the grid cell. Represents the center of the cell.	Min: −123.98 Max: −122.98
data	salinity	practical salinity units (PSU)	The mean practical salinity of the samples within the grid cell. Calculated from conductivity and temperature.	Min: 0 Max: 42
data	conductivity	mS · cm^−1^	The mean sea water electrical conductivity for the grid cell as measured by the thermosalinograph.	Min: 0 Max: 70
data	temperature	°C	The mean sea water temperature for the grid cell measured by the thermosalinograph.	Min: −5 Max: 35
data	intake_temperature	°C	TWS-NNO Only: The mean sea water temperature for the grid cell measured by the intake thermometer. This thermometer is located close to the intake valve (∼1–2 m).	Min: −5 Max: 35
data	dissolved oxygen	µmol · kg^−1^	The mean dissolved oxygen of the grid cell. These data are converted from the original units of mL·L^−1^ using potential density calculated from the co-located thermosalinograph.	Min: 0 Max: 500
data	oxygen_saturation	%	The mean dissolved oxygen saturation of the grid cell.	Min: 0 Max: 200
data	density	kg · m^−3^	The mean sea water density for the grid cell propagated from practical salinity and temperature from the thermosalinograph.	Min: 999 Max: 1038
**statistics Group**				
dimension	time	minutes since 1970–01–01	The approximate time a ferry departed a terminal.	Min: 2001–12–11T00:00:00Z
dimension	latitude	°N (decimal degrees)	The latitude of the grid cell. Represents the center of the cell.	Min: 48.67 Max: 49.26
dimension	longitude	°E (decimal degrees)	The longitude of the grid cell. Represents the center of the cell.	Min: −123.98 Max: −122.98
dimension	percentile	None	The percentile value for the corresponding *_percentile variable.	Values: 0, 5, 50, 95, 100
information	grid_time	seconds since 1970–01–01	The mean time of the samples that make up the grid cell.	Min: 2001–12–11T00:00:00Z
information	transit_end	minutes since 1970–01–01	The approximate time the ferry arrived at a terminal	Min: 2001–12–11T00:00:00Z
information	departure	None	Ferry departure location.	Values: TWS, NNO, SWB
information	arrival	None	Ferry arrival location.	Values: TWS, NNO, SWB
statistic	*_count	counts	The number of observations used to calculate the mean of the grid cell for the parent variable.	Not Applicable
statistic	*_std	Same as parent variable.	The standard deviation of the grid cell for the parent variable.	Not Applicable
statistic	*_sem	Same as parent variable.	The standard error of the mean (SEM) of the grid cell for the parent variable.	Not Applicable
statistic	*_percentile	Same as parent variable.	The percentile value of the grid cell for the parent variable.	Not Applicable

Footnote: * indicates the name of a corresponding parent variable. For example, the variable containing the standard error of the mean for temperature is temperature_sem. Valid min/max values represent the calibrated or valid range of values from each instrument or processing algorithm.

It should be noted that the data products compiled and calculated in this work are not the only measurements made available from the ferries. Measurements of fluorometric chlorophyll-a, fluorometric dissolved organic matter, turbidity, and the partial pressure of carbon dioxide in seawater are also available, but their quality is observed to be significantly influenced by system design, biofouling, and sediment accumulation between system cleanings, requiring careful review by end users. A variety of atmospheric and radiometric measurements captured aboard each ferry are also available but are not compiled here because their measurement is more continuous across time and not restricted by vessel motion.

## 4 Experimental Design, Materials and Methods

### 4.1 Data access and pre-processing

ONC hosts the historical data collected by the DFO and STRATOGEM ferry projects while also acting as the provider/maintainer for data collected after 2012. All raw and quality-assessed navigational, status, and science data were requested through the ONC Oceans Representational State Transfer (REST) Application Programming Interface (API) using the *onc* Python package [4] and repackaged into netCDF files.

Each individual measurement is accompanied by a quality flag that is generated from a set of automated quality assessment and control (QAQC) tests or it may be assigned by a human-in-the-loop (HITL) (see Table 1 in Owens et al. 2022 [3] for a description of ONC test criteria and flag meanings). As a precursory step, samples identified as having “bad” quality (flag = 4) were discarded. This flag indicates that a value fails a core set of primary tests (such as the gross range test) or that it was taken during a period when it would not have represented the environment. Latitude and longitude were then linearly interpolated with a maximum gap of 60 s to provide each sample with a position. Measurements taken within 250 m of known BCF terminals were discarded to help identify when each ferry was in transit and not at a terminal, as modern deployments continuously sample despite the system being closed off from the environment.

### 4.2 DFO (2001–2011) and STRATOGEM Project Data (2003–2006)

Data collected during the DFO and STRATOGEM projects are distributed as 30-second bin averages. STRATOGEM data are distributed with a quality assessment flag indicating that they were previously reviewed and binned by a human-in-the-loop (flag = 7). Data from the DFO project were quality assessed by ONC through automated tests, a HITL, and were pre-processed as previously described.

### 4.3 ONC Data (2012–2025)

Some data collected between 2012–2014 were sampled at a rate of 0.1 Hz (1 sample every 10 s). Otherwise, data were generally collected at a rate of 1 Hz. The Pump and Valve Control System (PVCS) is a custom ONC system designed to automate underway data collection while the vessel is in motion and to shut the system down during port stops and freshwater cleaning cycles. The supporting data for this system was not accessible prior to 2018.

Data collected after 2018 were and continue to be sampled at 1 Hz. In 2018, both the SoVI and QoA received PVCS updates that provided end users with key engineering (meta)data for assessing science data validity, such as the state of the system, the pump condition, and the flow rate of the water through the system. Freshwater tanks were also installed, allowing for freshwater to pumped through sensors to help mitigate the effect of biofouling between service and maintenance trips. With this new information, users may more readily separate original data that represents the environment from data captured when system was shut down or in a cleaning mode. The PVCS variables of system state, valve position, and pump current were linearly interpolated with a 30 s maximum gap. Data were then discarded if they met the following criteria:

• System state did not equal 1. System state of 0 indicates the system was disabled.
• Valve position did not equal 1. Valve position of 0 indicates closed and 2 indicates a freshwater flush.
• Pump current was <0.5A. Typical pump current draw is between 2–3 A. Data were discarded under the assumption that the pump was not providing optimal flow.

### 4.4 Computation of Additional Data Products

Prior to transit processing and spatial binning, several data products were computed following best practices (Table 2). To compute these, ancillary data were linearly interpolated along time with a maximum gap of 90 s.

**Table 2:** Methods for computed data products.

Variable	Ancillary Variables	Methodology/Reference	Description
salinity	conductivity, temperature	TEOS-10/GSW [5]	Practical salinity is calculated from conductivity and temperature using the gsw.SP_from_C function.
density	salinity, temperature	TEOS-10/GSW [5]	Density is calculated from absolute salinity and conservative temperature (from the base products of salinity and temperature) using the gsw.rho function.
dissolved_oxygen	salinity, temperature	Unit conversion, TEOS-10/GSW [5]	Dissolved oxygen is converted to µmol·kg^−1^ from mL·L^−1^ using potential density. Potential density is calculated from salinity and temperature to a reference pressure of 0 dbar (via gsw.pot_rho_t_exact).
oxygen_saturation	dissolved_oxygen, salinity, temperature	Garcia and Gordon 1992 [8], Benson and Krause, 2004 [9]	Oxygen saturation is estimated as a percentage with reference to equilibrium with the atmosphere (supported by gsw.O2sol).

Footnote: Computations requiring a pressure value used 3 dbar.

### 4.5 Individual Transit Processing

With the removal of port data, transits were separated assuming a minimum gap of 10 min between transits. Departure and arrival terminals were identified using the minimum geodesic distance to each known BCF terminal within the region. In the case of the TSW-NNO route, the arrival port periodically switched between the Duke Point and Departure Bay terminals (both in the Nanaimo area) and was generalized to NNO. Transits with time lengths <30 min and with the same departure and arrival terminals have been excluded in this curated dataset.

The modern version of the PVCS system closes the intake and outlet valve as the vessel approaches and remains at a ferry terminal. This inevitably leaves water to sit still within the system between transits and needs to be flushed before the system can sample new surface ocean water. Artificial warming by the vessel internal temperature is a strong indicator of stagnant water residing within the underway system and may be observed at the start of most transits in the original data (Fig. 2). These periods are most apparent within the first 60 s of the transit, regardless of terminal of origin, and are at risk of being mistaken for real oceanographic features.

**Fig. 2:**
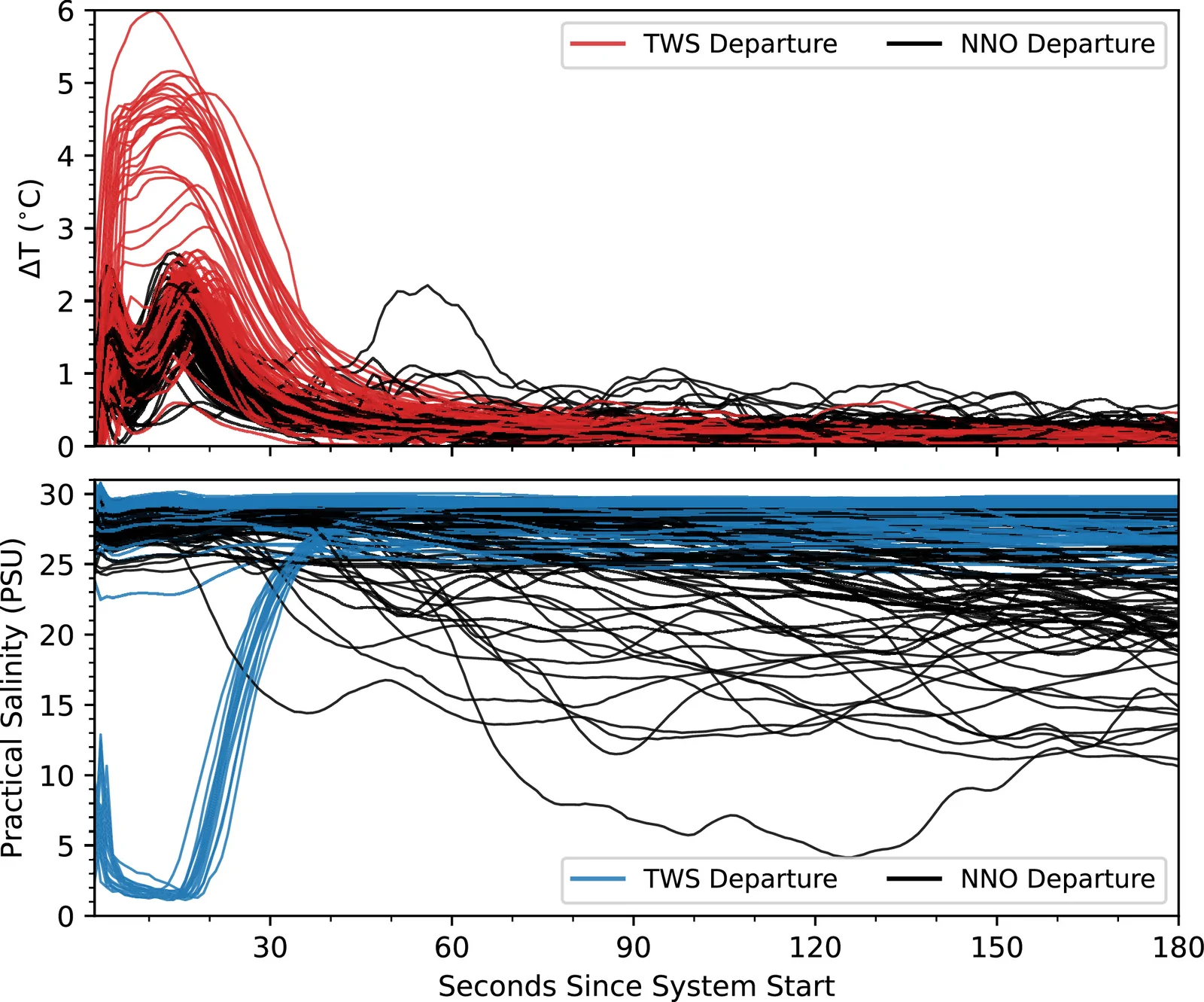
Example of stagnant water residing in the underway flowthrough system. Data are from 184 transits that occurred in March 2025. ∆T indicates the difference in temperature between the thermosalinograph and the intake thermometer (TSG-TEMPSENSOR). Lines are colored by the departure terminal. Note that the low salinity at the beginning of some transits is the result of the freshwater flush that occurs prior to the system shutting down while the vessel remains at Tsawwassen overnight. These overnight periods are also represented by the large differences in the two temperature measurements.

For transits that had an intake thermometer and thermosalinograph, both temperature measurements visually appear to stabilize and co-vary within 60 s of the sampling cycle start (Fig. 2), despite a notable lag between the measurements. At the time of curation, these data were being re-evaluated by ONC and may arrive with flags indicating OK (flag = 1) or suspect (flag = 3) quality. Typical flow rates range between 3–5 L·min^−1^ and the system volume on each vessel can be approximated as 3–4 L. From this empirical assessment and as a safety margin, the first 150 s of each transit have been discarded in the curated route datasets under the assumption that these data largely represented stagnant-mixed water within the underway flowthrough system and to allow sufficient time for new surface water to be observed.

The sampling rate for each instrument has also varied at each system design iteration. For transits where the sampling rate was 1 Hz, a median filter with a window size of 7 was applied to help remove abrupt spikes. Archived transits with slower sampling rates had no signal filters applied. Data were then compiled into 0.01°x0.01° bins and the beginning timestamp of the transit was assigned as the unique transit identifier. Fig. 3, Fig. 4, Fig. 5 provide visual representation examples of the gridded data as individual transit periods and over time.

**Fig. 3:**
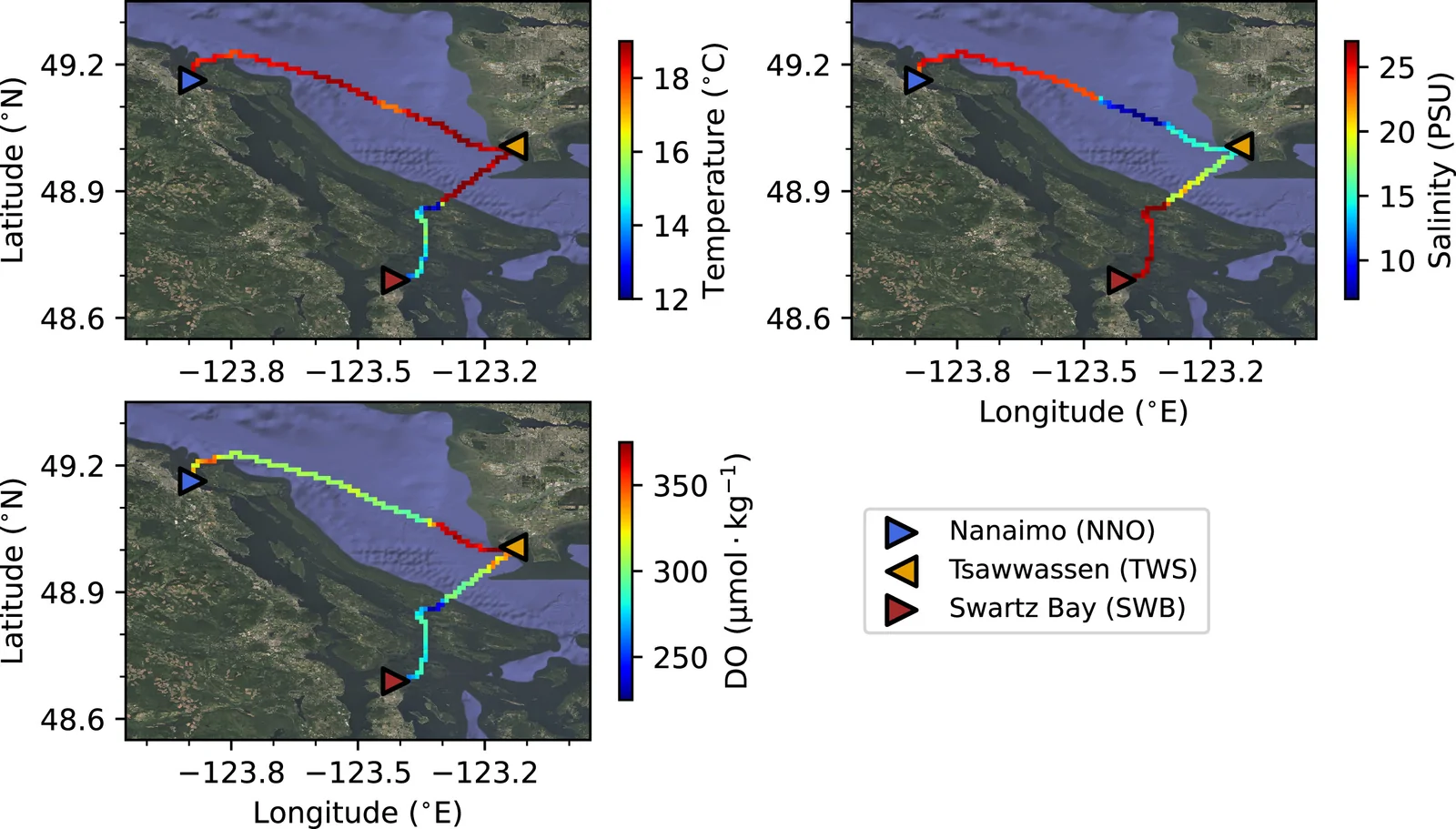
Example of two contemporary transits. Both transits began around 1810 UTC on 2025–07–04, with the vessel on the TWS-NNO route departing from TWS and the vessel on the TWS-SWB route departing from SWB. This figure highlights differences in the physical characteristics between the northern Strait of Georgia, Fraser River plume, and southern Strait of Georgia. Imagery ©2026 NASA, Map Data ©2026 Google.

**Fig. 4:**
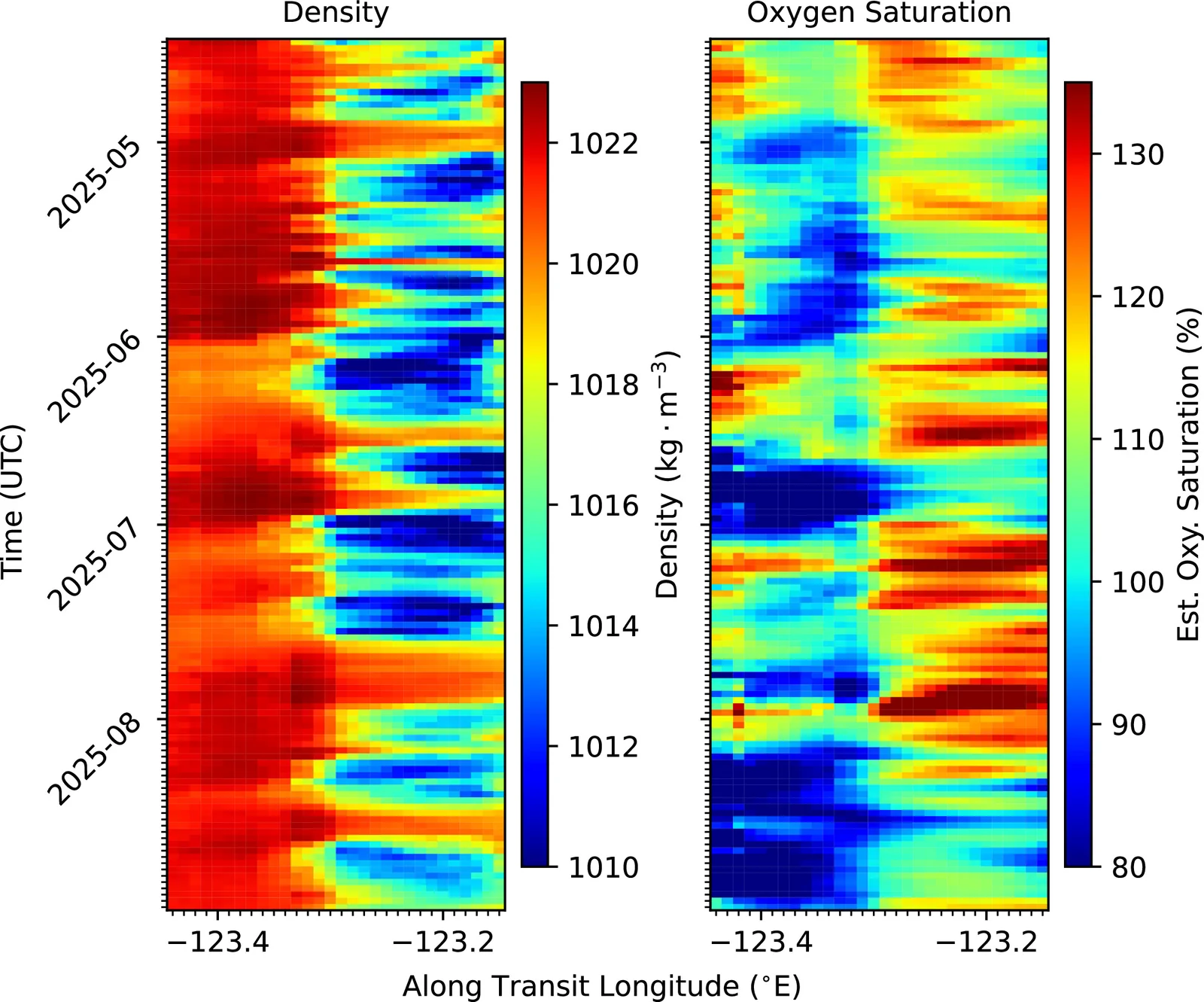
Example Hovmöller plot of density and oxygen saturation along the TWS-SWB route for a subset of data in 2025. In this figure, data were first averaged over latitude and then by 1-day periods. This figure highlights relationships between the physical characteristics of water and oxygen content along the TWS-SWB route.

**Fig. 5:**
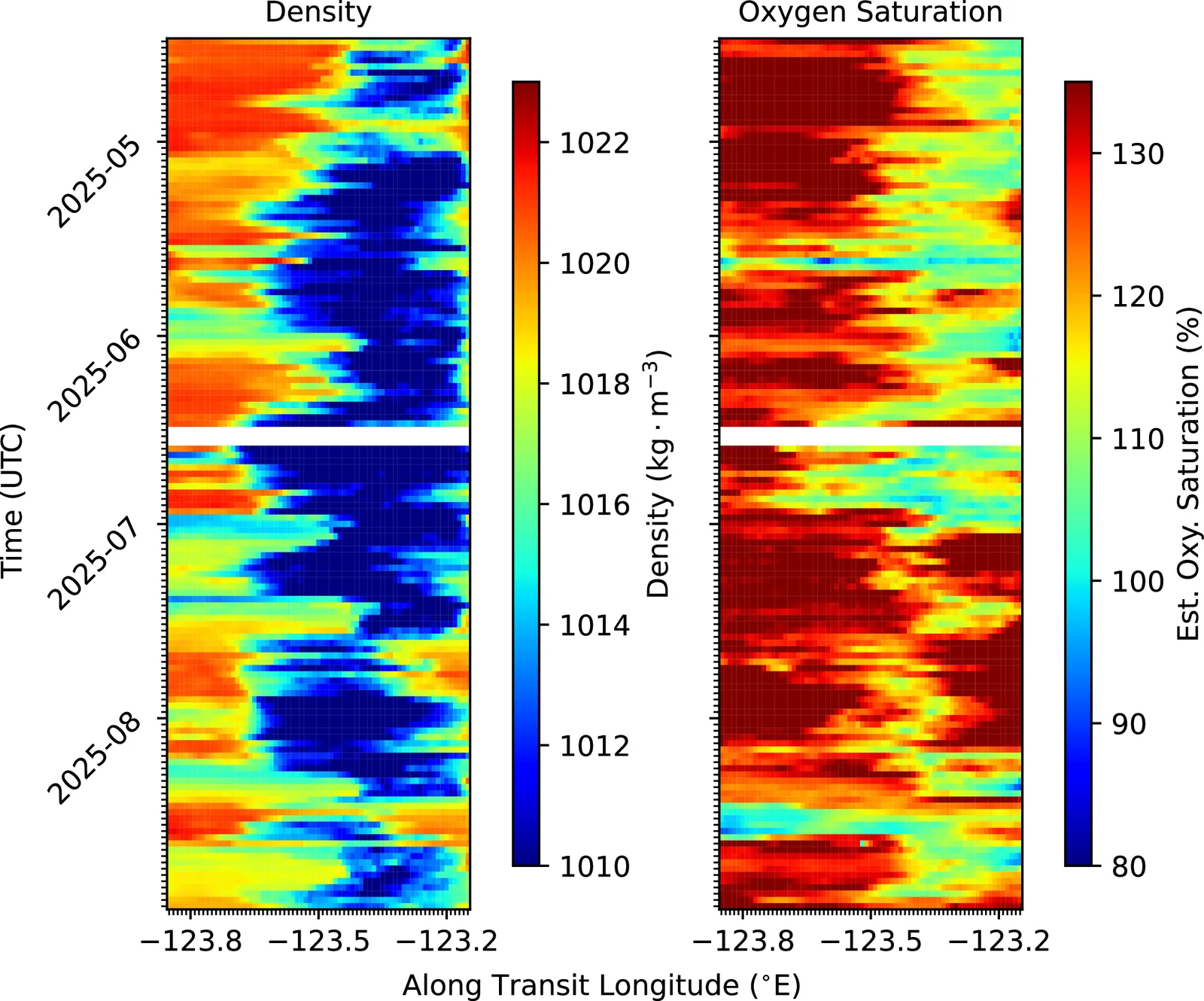
Example Hovmöller plot of density and oxygen saturation along the TWS-NNO route for a subset of data in 2025. In this figure, data were first averaged over latitude and then by 1-day periods. This figure highlights relationships between the physical characteristics of water and oxygen content along the TWS-NNO route.

## 5 Limitations

### 5.1 Data Continuity

Data are unfortunately not continuous between 2001–2025. Fig. 6 visualizes the gaps in each available data product for each route and the density of transits passing through grid cells in the region. Gaps in data either occurred due to system downtime or because the ferry temporarily moved to assist with another BCF route. For data products like dissolved oxygen and estimated oxygen saturation, the availability of those data is also limited by the contemporary installation of instruments that measure the ancillary variables in Table 2. For finer coverage of data availability, users may use the ONC DataSearch Tool (https://data.oceannetworks.ca/DataSearch) or a Hierarchical Data Format (HDF) file tool to review the curated netCDF files.

**Fig. 6:**
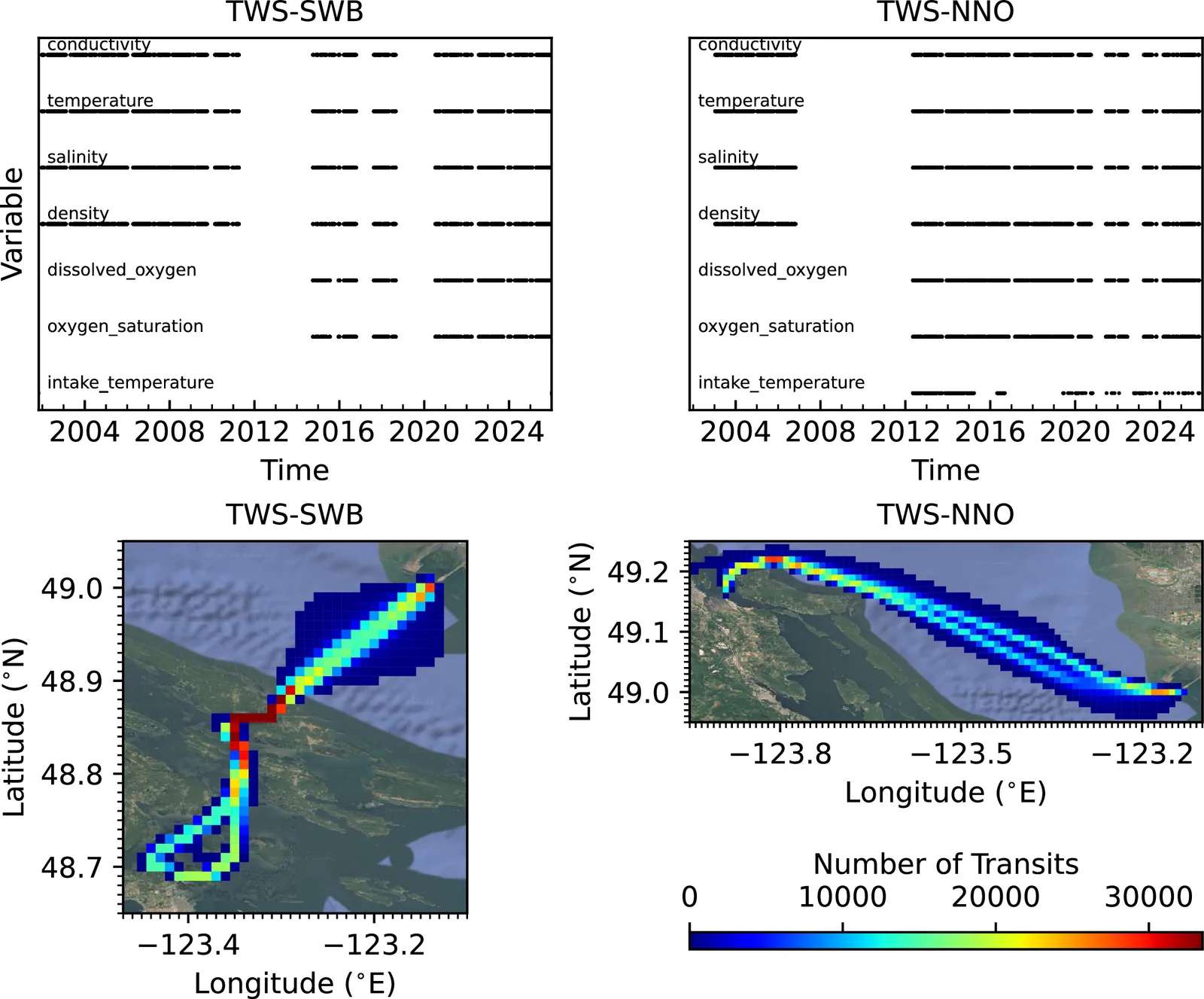
Data availability by variable along each route. The top subplots represent the series of days that have at least one transit that collected that data product. The bottom subplots represent the number of transits that have occurred within a particular grid cell for each route based on the availability of temperature data. Imagery ©2026 NASA, Map Data ©2026 Google.

### 5.2 Uncertainty in Measurements

The base and drift uncertainty associated with an instrument is not always reported consistently by the manufacturer and may appear as terms such as accuracy, precision, sensitivity, or stability [6]. This value is different from the standard error of the mean (SEM), which may be used to indicate uncertainty with the fluctuation of the measurement over a defined bin or window [7]. The root square sum of the instrument accuracy and the SEM may then be used to provide an overall estimate of the uncertainty (see Eq. 2 of Waldmann et al. 2022) [7]. Table 3 provides the base measurement uncertainties and estimates of drift for the variables in this curated dataset identified in available manuals.

**Table 3:** Measurement uncertainties.

Instrument Measurement	Estimated Base Uncertainty	Estimated Drift
PT-100 RTD Temperature	±0.05 °C	Depends on RTD and electronics quality. ±0.02 °C to ±0.05 °C per month
SBE-21 Thermosalinograph Temperature	±0.01 °C	±0.002 °C per month [11]
SBE-21 Thermosalinograph Conductivity	±0.01 mS · cm^−1^	±0.0015 mS cm^−1^ per month [11]
SBE-38 Digital Thermometer Temperature	±0.001 °C	±0.00033 °C per month
SBE-45 Thermosalinograph Temperature	±0.002 °C	±0.002 °C per month
SBE-45 Thermosalinograph Practical Salinity	±0.005 PSU	±0.003 PSU per month
Aanderraa 3835 Dissolved Oxygen	5%	Time/Sample Dependent, Exponential Decay, Improves Over Sample Time [10]

Footnote: PT-100 drift estimates vary from manufacturer to manufacturer. We provide an approximate range based on low-cost PT-100 thermistors.

Identifying and estimating drift is a significant limitation in assessing the quality of the data produced by the ferries. For long-term identification, users may consider plotting a variable with respect to grid_time or using the original data to identify long-term drift trends. In general, instruments deployed on the ferries are recalibrated at the factory every 2–3 years. For a first order drift estimate, users may consider a linear interpolation over time from the time the instrument was calibrated to the time it was recovered. This information can be identified through a combination of ONC’s Annotation Search service (https://data.oceannetworks.ca/AnnotationsV2) and Device log (https://data.oceannetworks.ca/app/devices).

### 5.3 Temperature Change in Flowthrough

The difference in temperature between the intake thermometer and thermosalinograph was used to approximate the amount of time it takes the QoA underway system to begin to sample new water after sequence startup. Generally, this change in temperature indicates warming imparted by the vessel and users may observe a semi-regular offset. The computation of salinity, density and correction of dissolved oxygen are all sensitive to temperature, but unfortunately the difference in temperature cannot be computed across the entire TWS-NNO dataset or for the TWS-SWB dataset. The underway flowthrough systems have been installed in similar locations on each vessel, so users may be inclined to recalculate temperature sensitive products using existing intake temperature data or by assuming a static or time-varying offset.

### 5.4 Validation

These curated data have not been assessed extensively against external data sources. The availability of open and accessible surface measurements co-located with each ferry route are limited in both space and time. The Canada Integrated Ocean Observing System Data Explorer (CIOOS, https://explore.cioos.ca/) is an example of a resource that may be utilized to obtain publicly available discrete samples and instrument measurements. For example, sampling campaigns from the DFO collected discrete samples and vertical profiles along the TWS-NNO route, but coverage within CIOOS is limited, offering comparison for only a few ferry instrument deployments.

Remote reflectance at 555 nm may be used to infer turbid river plumes along coastlines (e.g. Saldías et al. 2016 [12]). Visually, spatial agreement may be inferred between the edges of the turbid river plume observed with Rrs_555_ and sea surface density calculated from ferry data (Fig. 7).

**Fig. 7:**
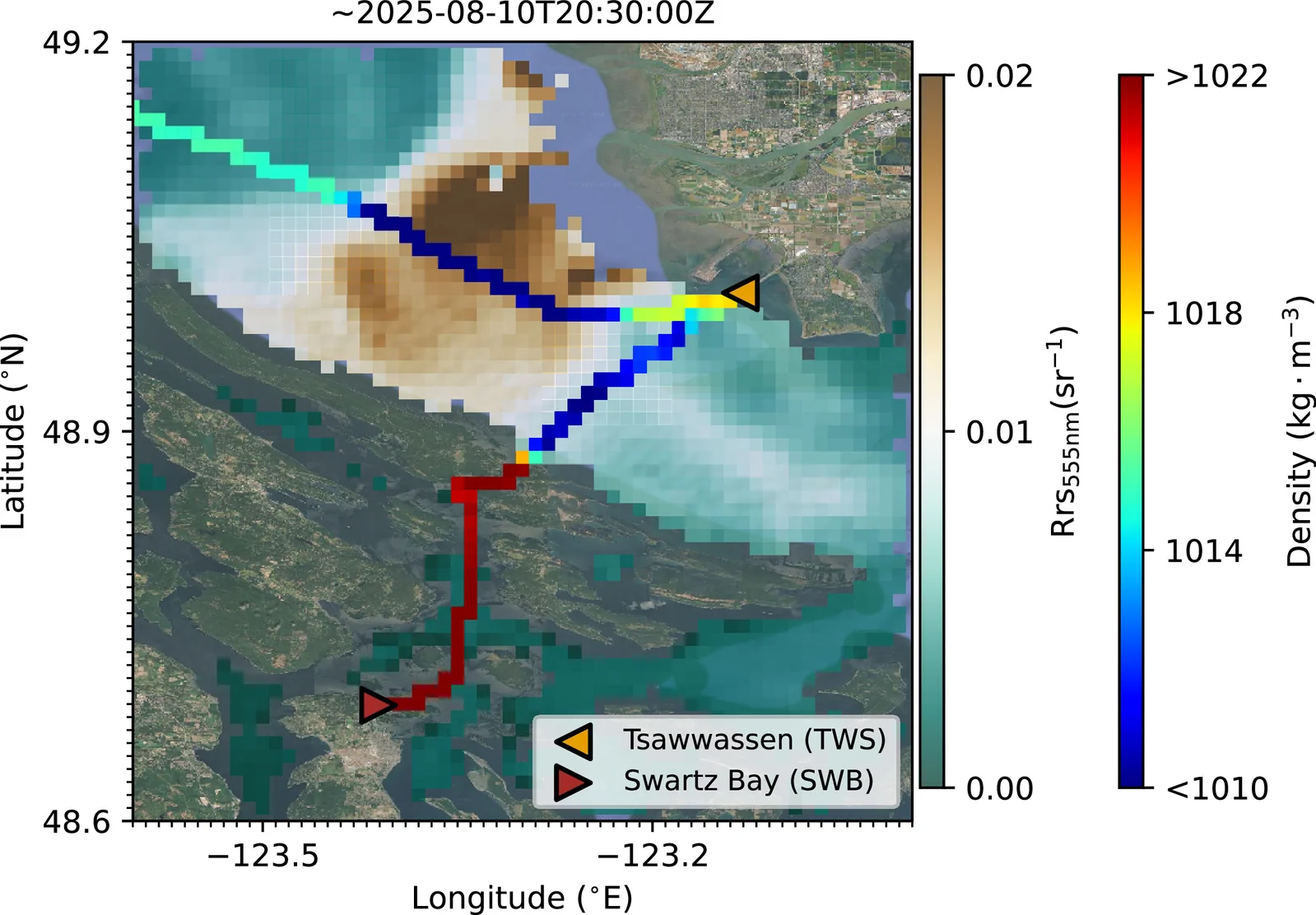
Sea surface density from the ferries overlaying Rrs_555_ for the southern Strait of Georgia, 2025–08–10. Rrs_555_ is gridded to 0.01° from NASA PACE OCI Level 2 Apparent Optical Properties V3.2 (PACE_OCI.20250810T203046.L2.OC_AOP.V3_2.nc) [13]. Ferry data are from transits that occurred nearest to the PACE OCI file time (within 1 hour). Imagery ©2026 NASA, Map Data ©2026 Google.

Comparison between routes is possible near Tsawwassen and the Concordance Correlation Coefficient (ρ_c_) is one metric which may be used to infer agreement between two sampling methods [14]. Comparison between the two routes across the time-series shows excellent agreement between temperature measurements (ρ_c_ = 0.98, n = 2008), moderate agreement between salinity measurements (ρ_c_ = 0.85, n = 2005), and weak agreement between dissolved oxygen measurements (ρ_c_ = 0.5, n = 1669). Users should note large differences at extremes (Fig. 8) and consider instrument accuracy, system design, and seasonal/environmental influences (e.g. plume-driven fronts, tides, winds, etc.) when assessing drivers for non-zero differences between each route and when comparing to other data products.

**Fig. 8:**
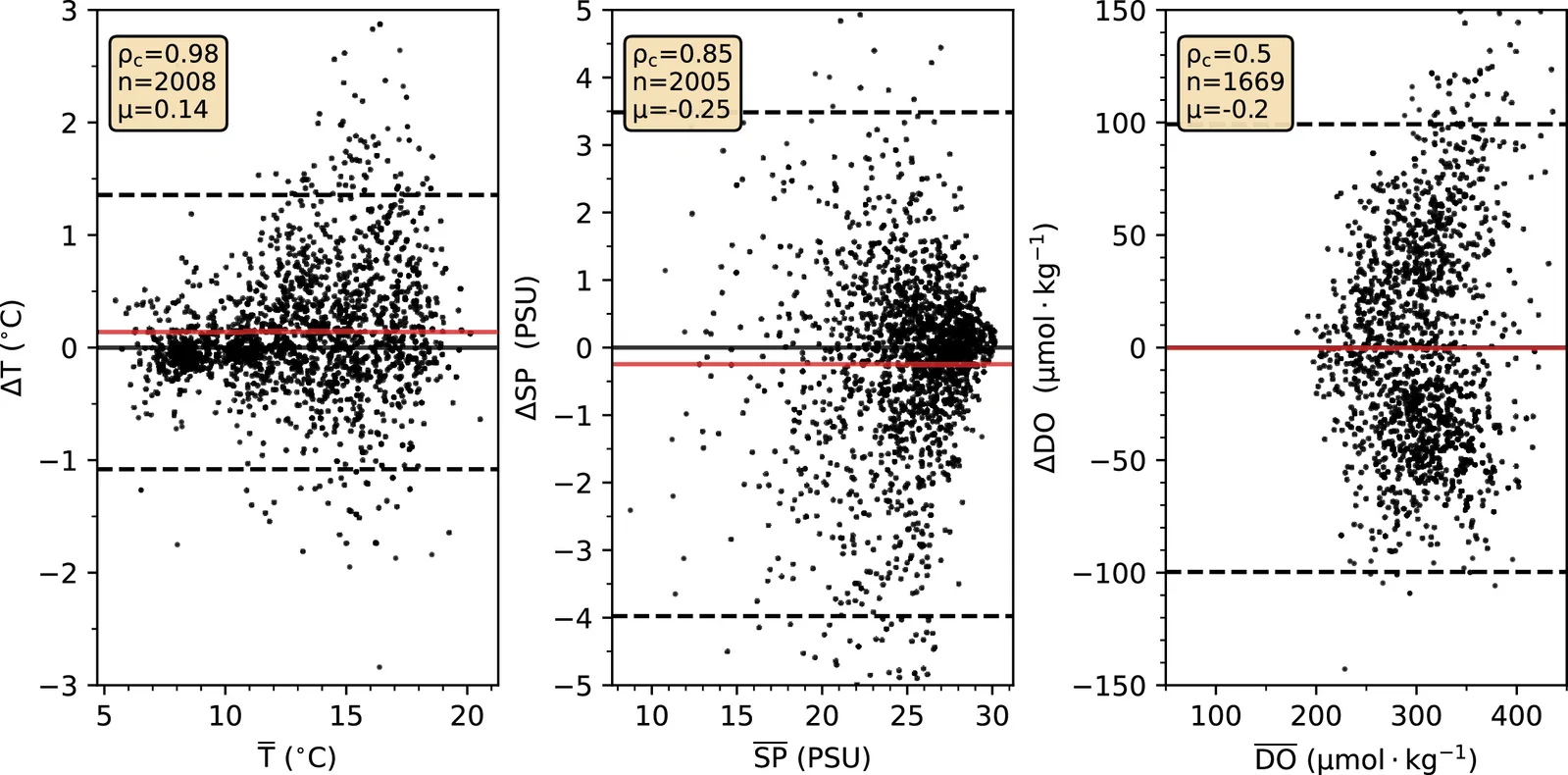
Bland-Altman plots for temperature, salinity, and dissolved oxygen differences between TWS-SWB and TWS-NNO. T indicates temperature, SP indicates practical salinity, and DO indicates dissolved oxygen. ρ_c_ is the Concordance Correlation Coefficient. Tsawwassen arrival transits occurring within 60 min of each other and the mean of a 0.03° box near Tsawwassen are used for comparison (>1600 instances between 2003–2025). The vertical axis represents the difference between the measurements captured by the TWS-SWB and TWS-NNO (TWS-SWB minus TWS-NNO). The horizontal axis represents the mean of the two measurements. The mean bias (µ, red horizontal line) for TWS-SWB - TWS-NNO is 0.14, −0.25, and −0.2 for temperature, salinity, and dissolved oxygen, respectively. The dashed black lines indicate the upper and lower limits of agreement (µ ± 1.96σ).

## Ethics Statement

The authors have reviewed the ethical requirements for publication in Data in Brief and confirm that this work does not involve human subjects, animal experiments, or any data collected from social media platforms.

## CRediT Author Statement

ITB: Conceptualization, Methodology, Software, Formal Analysis, Data Curation, Writing -Original Draft, Visualization

AOVB: Validation, Investigation, Data Curation, Writing–Review & Editing

BLM: Formal Analysis, Investigation, Writing–Review & Editing

KWB: Writing–Review & Editing, Supervision, Project Administration, Resources

## Original Data Citations

Ocean Networks Canada Society. 2011. Tsawwassen - Duke Point Ferry Route Thermosalinograph Deployed 2003–01–13. Ocean Networks Canada Society. https://doi.org/10.34943/c6b0b23f-b60b-4ba9-a7ba-de0c68130c0d. Accessed 2026–05–16.

Ocean Networks Canada Society. 2011. Tsawwassen - Duke Point Ferry Route Thermosalinograph Deployed 2003–03–11. Ocean Networks Canada Society. https://doi.org/10.34943/3d35eca8-70cf-45ce-aea2-98833d31e62c. Accessed 2026–05–16.

Ocean Networks Canada Society. 2011. Tsawwassen - Duke Point Ferry Route Thermosalinograph Deployed 2003–05–09. Ocean Networks Canada Society. https://doi.org/10.34943/b44bf38b-8fb2-43c1-b920-bdfd7a2ceee0. Accessed 2026–05–16.

Ocean Networks Canada Society. 2011. Tsawwassen - Duke Point Ferry Route Thermosalinograph Deployed 2003–12–23. Ocean Networks Canada Society. https://doi.org/10.34943/98f5ebe8-5911-4828-86ab-c9bc853f332e. Accessed 2026–05–16.

Ocean Networks Canada Society. 2011. Tsawwassen - Duke Point Ferry Route Thermosalinograph Deployed 2005–01–06. Ocean Networks Canada Society. https://doi.org/10.34943/634edcc6-59ef-469a-a52a-d0a5e7fe7107. Accessed 2026–05–16.

Ocean Networks Canada Society. 2012. Tsawwassen - Duke Point Ferry Route Navigation System Deployed 2012–05–06. Ocean Networks Canada Society. https://doi.org/10.34943/2f8361d2-36a7-4933-a899-f2bd8d9800fa. Accessed 2026–05–16.

Ocean Networks Canada Society. 2012. Tsawwassen - Duke Point Ferry Route Oxygen Sensor Deployed 2012–05–06. Ocean Networks Canada Society. https://doi.org/10.34943/9df11545-85db-4422-9a13-ce73eea30c8b. Accessed 2026–05–16.

Ocean Networks Canada Society. 2012. Tsawwassen - Duke Point Ferry Route Temperature Sensor Deployed 2012–05–06. Ocean Networks Canada Society. https://doi.org/10.34943/95089573-3863-4385-a96f-60d62d606ca8. Accessed 2026–05–16.

Ocean Networks Canada Society. 2012. Tsawwassen - Duke Point Ferry Route Thermosalinograph Deployed 2012–05–06. Ocean Networks Canada Society. https://doi.org/10.34943/73e5fa18-987f-4555-8664-d2b36f354bef. Accessed 2026–05–16.

Ocean Networks Canada Society. 2012. Tsawwassen - Duke Point Ferry Route Thermosalinograph Deployed 2012–08–03. Ocean Networks Canada Society. https://doi.org/10.34943/16bed33d-6a47-46bc-a6cb-fd90891f95e9. Accessed 2026–05–16.

Ocean Networks Canada Society. 2013. Tsawwassen - Duke Point Ferry Route Oxygen Sensor Deployed 2013–11–08. Ocean Networks Canada Society. https://doi.org/10.34943/a1dfff01-68a4-41e1-971f-ec5697525a86. Accessed 2026–05–16.

Ocean Networks Canada Society. 2013. Tsawwassen - Duke Point Ferry Route Thermosalinograph Deployed 2013–11–08. Ocean Networks Canada Society. https://doi.org/10.34943/227a4acd-0291-4d87-aaad-a9b1a1769a63. Accessed 2026–05–16.

Ocean Networks Canada Society. 2014. Tsawwassen - Swartz Bay Ferry Route Navigation System Deployed 2014–10–02. Ocean Networks Canada Society. https://doi.org/10.34943/fbaa9b17-6ef9-4005-bfff-c0361c2b9bf4. Accessed 2026–05–16.

Ocean Networks Canada Society. 2014. Tsawwassen - Swartz Bay Ferry Route Oxygen Sensor Deployed 2014–10–02. Ocean Networks Canada Society. https://doi.org/10.34943/06d2afa2-ea04-4ec1-aa80-05e00c15f4e3. Accessed 2026–05–16.

Ocean Networks Canada Society. 2014. Tsawwassen - Swartz Bay Ferry Route Thermosalinograph Deployed 2014–10–02. Ocean Networks Canada Society. https://doi.org/10.34943/5ea7a877-49ad-4e5a-8146-fcd2ae46b158. Accessed 2026–05–16.

Ocean Networks Canada Society. 2015. Tsawwassen - Duke Point Ferry Route Oxygen Sensor Deployed 2015–01–09. Ocean Networks Canada Society. https://doi.org/10.34943/831e0e27-f6f3-4bf2-84f6-7c7236a89ce5. Accessed 2026–05–16.

Ocean Networks Canada Society. 2015. Tsawwassen - Duke Point Ferry Route Oxygen Sensor Deployed 2015–11–13. Ocean Networks Canada Society. https://doi.org/10.34943/c8e88859-4c14-41ff-bf34-8d6723ee6fd5. Accessed 2026–05–16.

Ocean Networks Canada Society. 2015. Tsawwassen - Duke Point Ferry Route Thermosalinograph Deployed 2015–11–13. Ocean Networks Canada Society. https://doi.org/10.34943/04fc1839-683f-4065-912c-40451947fba3. Accessed 2026–05–16.

Ocean Networks Canada Society. 2015. Tsawwassen - Swartz Bay Ferry Route Oxygen Sensor Deployed 2015–11–19. Ocean Networks Canada Society. https://doi.org/10.34943/d0ed1cd0-10a4-45fc-b75a-5e65c522db9e. Accessed 2026–05–16.

Ocean Networks Canada Society. 2016. Tsawwassen - Duke Point Ferry Route Temperature Sensor Deployed 2016–02–02. Ocean Networks Canada Society. https://doi.org/10.34943/380f2289-1b64-449b-ac85-33dd2d8062fc. Accessed 2026–05–16.

Ocean Networks Canada Society. 2016. Tsawwassen - Swartz Bay Ferry Route Oxygen Sensor Deployed 2016–02–03. Ocean Networks Canada Society. https://doi.org/10.34943/97325117-8ddf-4a09-a274-9c2ea8bd172f. Accessed 2026–05–16.

Ocean Networks Canada Society. 2016. Tsawwassen - Swartz Bay Ferry Route Thermosalinograph Deployed 2016–02–03. Ocean Networks Canada Society. https://doi.org/10.34943/69ef7f6c-9c49-445a-8b2e-1a4dbceff497. Accessed 2026–05–16.

Ocean Networks Canada Society. 2017. Tsawwassen - Duke Point Ferry Route Navigation System Deployed 2017–02–15. Ocean Networks Canada Society. https://doi.org/10.34943/6225b542-2c90-414d-85b6-d2b9c3765d56. Accessed 2026–05–16.

Ocean Networks Canada Society. 2017. Tsawwassen - Duke Point Ferry Route Navigation System Deployed 2017–02–15. Ocean Networks Canada Society. https://doi.org/10.34943/b3997620-557f-4bb4-b85f-f1b20c002619. Accessed 2026–05–16.

Ocean Networks Canada Society. 2017. Tsawwassen - Duke Point Ferry Route Navigation System Deployed 2017–03–01. Ocean Networks Canada Society. https://doi.org/10.34943/ec72c0d1-731b-4a25-a0f9-a3be042266a6. Accessed 2026–05–16.

Ocean Networks Canada Society. 2017. Tsawwassen - Duke Point Ferry Route Oxygen Sensor Deployed 2017–02–15. Ocean Networks Canada Society. https://doi.org/10.34943/27157169-8813-4356-8a28-a3304b868a24. Accessed 2026–05–16.

Ocean Networks Canada Society. 2017. Tsawwassen - Duke Point Ferry Route Thermosalinograph Deployed 2017–02–15. Ocean Networks Canada Society. https://doi.org/10.34943/e67fd589-1cbf-4253-b0dc-febac860067f. Accessed 2026–05–16.

Ocean Networks Canada Society. 2017. Tsawwassen - Swartz Bay Ferry Route Navigation System Deployed 2017–08–15. Ocean Networks Canada Society. https://doi.org/10.34943/5d7c08b1-63df-4b84-987e-a3b336607d59. Accessed 2026–05–16.

Ocean Networks Canada Society. 2017. Tsawwassen - Swartz Bay Ferry Route Oxygen Sensor Deployed 2017–08–15. Ocean Networks Canada Society. https://doi.org/10.34943/17ad76f3-430f-44e9-b644-aff5575a0672. Accessed 2026–05–16.

Ocean Networks Canada Society. 2017. Tsawwassen - Swartz Bay Ferry Route Pump and Valve Control System Deployed 2017–08–15. Ocean Networks Canada Society. https://doi.org/10.34943/f934709c-ab1d-4018-b6f0-de3b7ade21cc. Accessed 2026–05–16.

Ocean Networks Canada Society. 2017. Tsawwassen - Swartz Bay Ferry Route Thermosalinograph Deployed 2017–08–15. Ocean Networks Canada Society. https://doi.org/10.34943/970f127b-13ba-4b5c-9ac5-f5e9797e711e. Accessed 2026–05–16.

Ocean Networks Canada Society. 2018. Tsawwassen - Duke Point Ferry Route Oxygen Sensor Deployed 2018–08–08. Ocean Networks Canada Society. https://doi.org/10.34943/92680252-756c-473d-ab10-284ee4d1cbd5. Accessed 2026–05–16.

Ocean Networks Canada Society. 2018. Tsawwassen - Duke Point Ferry Route Oxygen Sensor Deployed 2018–12–19. Ocean Networks Canada Society. https://doi.org/10.34943/8c6acff7-6a14-4c4a-9804-f00bfde9675e. Accessed 2026–05–16.

Ocean Networks Canada Society. 2018. Tsawwassen - Duke Point Ferry Route Pump and Valve Control System Deployed 2018–04–26. Ocean Networks Canada Society. https://doi.org/10.34943/6a525113-3a5a-4dc8-bc58-8ffdf7a3cc8d. Accessed 2026–05–16.

Ocean Networks Canada Society. 2018. Tsawwassen - Duke Point Ferry Route Temperature Sensor Deployed 2018–12–05. Ocean Networks Canada Society. https://doi.org/10.34943/cab1196d-1366-42fa-99f2-6cb5485e4a9c. Accessed 2026–05–16.

Ocean Networks Canada Society. 2019. Tsawwassen - Duke Point Ferry Route Oxygen Sensor Deployed 2019–10–03. Ocean Networks Canada Society. https://doi.org/10.34943/4c0ad657-4e97-4ae7-9c56-7109d7345d1d. Accessed 2026–05–16.

Ocean Networks Canada Society. 2019. Tsawwassen - Duke Point Ferry Route Thermosalinograph Deployed 2019–11–08. Ocean Networks Canada Society. https://doi.org/10.34943/20fdaf30-ba61-4e19-80b9-045f27020127. Accessed 2026–05–16.

Ocean Networks Canada Society. 2020. Tsawwassen - Swartz Bay Ferry Route Navigation System Deployed 2020–02–19. Ocean Networks Canada Society. https://doi.org/10.34943/650bbbf7-c3ab-4fb5-9980-5730dfa839c3. Accessed 2026–05–16.

Ocean Networks Canada Society. 2020. Tsawwassen - Swartz Bay Ferry Route Oxygen Sensor Deployed 2020–05–20. Ocean Networks Canada Society. https://doi.org/10.34943/60de0dde-466b-4059-9668-5de4cfd26646. Accessed 2026–05–16.

Ocean Networks Canada Society. 2020. Tsawwassen - Swartz Bay Ferry Route Pump and Valve Control System Deployed 2020–06–16. Ocean Networks Canada Society. https://doi.org/10.34943/97ee850e-6beb-4700-983e-e4e342b99599. Accessed 2026–05–16.

Ocean Networks Canada Society. 2020. Tsawwassen - Swartz Bay Ferry Route Thermosalinograph Deployed 2020–05–20. Ocean Networks Canada Society. https://doi.org/10.34943/b8c71c03-7b83-416d-a6e3-5e95b54e8524. Accessed 2026–05–16.

Ocean Networks Canada Society. 2021. Tsawwassen - Swartz Bay Ferry Route Oxygen Sensor Deployed 2021–07–12. Ocean Networks Canada Society. https://doi.org/10.34943/68bfd06b-0068-47d7-bbc1-f85da97db487. Accessed 2026–05–16.

Ocean Networks Canada Society. 2021. Tsawwassen - Swartz Bay Ferry Route Thermosalinograph Deployed 2021–07–12. Ocean Networks Canada Society. https://doi.org/10.34943/c5ccc7ee-1f5c-499b-a398-1afbf71b80fd. Accessed 2026–05–16.

Ocean Networks Canada Society. 2022. Tsawwassen - Duke Point Ferry Route Navigation System Deployed 2022–10–11. Ocean Networks Canada Society. https://doi.org/10.34943/18be8ece-e4b3-4882-91b1-41e3c899ef80. Accessed 2026–05–16.

Ocean Networks Canada Society. 2022. Tsawwassen - Duke Point Ferry Route Oxygen Sensor Deployed 2022–10–11. Ocean Networks Canada Society. https://doi.org/10.34943/6a1fcef3-f9c1-4102-a4a2-de10eb9eb218. Accessed 2026–05–16.

Ocean Networks Canada Society. 2022. Tsawwassen - Duke Point Ferry Route Pump and Valve Control System Deployed 2022–10–11. Ocean Networks Canada Society. https://doi.org/10.34943/b34c846b-e634-4e0c-8f15-d7ac5af079c5. Accessed 2026–05–16.

Ocean Networks Canada Society. 2022. Tsawwassen - Duke Point Ferry Route Temperature Sensor Deployed 2022–10–11. Ocean Networks Canada Society. https://doi.org/10.34943/3b7c0745-29a9-45aa-8a6e-12552762a7e3. Accessed 2026–05–16.

Ocean Networks Canada Society. 2022. Tsawwassen - Duke Point Ferry Route Thermosalinograph Deployed 2022–10–11. Ocean Networks Canada Society. https://doi.org/10.34943/e39bb1d9-fa54-4bf1-a18d-01437618b52b. Accessed 2026–05–16.

Ocean Networks Canada Society. 2023. Tsawwassen - Duke Point Ferry Route Oxygen Sensor Deployed 2023–01–13. Ocean Networks Canada Society. https://doi.org/10.34943/7c9b9896-328a-40dc-ae42-2a1fecca9133. Accessed 2026–05–16.

Ocean Networks Canada Society. 2023. Tsawwassen - Duke Point Ferry Route Thermosalinograph Deployed 2023–01–13. Ocean Networks Canada Society. https://doi.org/10.34943/be26b40e-9e90-41b4-b6ff-7bbf035847f4. Accessed 2026–05–16.

Ocean Networks Canada Society. 2023. Tsawwassen - Swartz Bay Ferry Route Oxygen Sensor Deployed 2023–05–23. Ocean Networks Canada Society. https://doi.org/10.34943/845ae618-4ca5-47e3-a250-6fe25acd3df5. Accessed 2026–05–16.

Ocean Networks Canada Society. 2024. Tsawwassen - Duke Point Ferry Route Navigation System Deployed 2024–06–20. Ocean Networks Canada Society. https://doi.org/10.34943/0b3f8b6a-9069-456f-b0d1-ce138f9e2a6c. Accessed 2026–05–16.

Ocean Networks Canada Society. 2024. Tsawwassen - Duke Point Ferry Route Navigation System Deployed 2024–10–01. Ocean Networks Canada Society. https://doi.org/10.34943/37f108d9-89d7-49b8-b677-770e0bc6068e. Accessed 2026–05–16.

Ocean Networks Canada Society. 2024. Tsawwassen - Duke Point Ferry Route Oxygen Sensor Deployed 2024–06–20. Ocean Networks Canada Society. https://doi.org/10.34943/7d9ed37f-281c-4197-b371-2fa67de38fcb. Accessed 2026–05–16.

Ocean Networks Canada Society. 2024. Tsawwassen - Duke Point Ferry Route Oxygen Sensor Deployed 2024–10–01. Ocean Networks Canada Society. https://doi.org/10.34943/e34f1236-b902-474a-8183-80c85a5681df. Accessed 2026–05–16.

Ocean Networks Canada Society. 2024. Tsawwassen - Duke Point Ferry Route Pump and Valve Control System Deployed 2024–06–20. Ocean Networks Canada Society. https://doi.org/10.34943/fe4fb32d-bd44-42c2-a8a6-66bf982a8a91. Accessed 2026–05–16.

Ocean Networks Canada Society. 2024. Tsawwassen - Duke Point Ferry Route Pump and Valve Control System Deployed 2024–10–01. Ocean Networks Canada Society. https://doi.org/10.34943/7446bf3e-d358-4899-9040-b5c3eac127c8. Accessed 2026–05–16.

Ocean Networks Canada Society. 2024. Tsawwassen - Duke Point Ferry Route Temperature Sensor Deployed 2024–06–20. Ocean Networks Canada Society. https://doi.org/10.34943/6d1f95e1-d6c0-43e5-8405-73a960b7e295. Accessed 2026–05–16.

Ocean Networks Canada Society. 2024. Tsawwassen - Duke Point Ferry Route Temperature Sensor Deployed 2024–10–01. Ocean Networks Canada Society. https://doi.org/10.34943/d7994814-c62b-4c61-a25d-95ba1322d16a. Accessed 2026–05–16.

Ocean Networks Canada Society. 2024. Tsawwassen - Duke Point Ferry Route Thermosalinograph Deployed 2024–06–20. Ocean Networks Canada Society. https://doi.org/10.34943/e59b9fdb-4226-4f9a-af50-94965ba4da1d. Accessed 2026–05–16.

Ocean Networks Canada Society. 2024. Tsawwassen - Duke Point Ferry Route Thermosalinograph Deployed 2024–10–01. Ocean Networks Canada Society. https://doi.org/10.34943/c3f14be1-0c62-4ef1-9df7-b8ce1b12e409. Accessed 2026–05–16.

Ocean Networks Canada Society. 2024. Tsawwassen - Swartz Bay Ferry Route Oxygen Sensor Deployed 2024–12–13. Ocean Networks Canada Society. https://doi.org/10.34943/3943c511-0e0d-4b3a-b9a2-1f739abcb484. Accessed 2026–05–16.

Ocean Networks Canada Society. 2025. Tsawwassen - Duke Point Ferry Route Navigation System Deployed 2025–03–20. Ocean Networks Canada Society. https://doi.org/10.34943/16640b95-1580-4efc-9a88-beacf8cf6b72. Accessed 2026–05–16.

Ocean Networks Canada Society. 2025. Tsawwassen - Duke Point Ferry Route Navigation System Deployed 2025–06–19. Ocean Networks Canada Society. https://doi.org/10.34943/bfcde592-ead6-4894-bd2f-0c470f2850fa. Accessed 2026–05–16.

Ocean Networks Canada Society. 2025. Tsawwassen - Duke Point Ferry Route Navigation System Deployed 2025–10–15. Ocean Networks Canada Society. https://doi.org/10.34943/029493d0-c5bd-4cfe-8e54-cfe216ad6046. Accessed 2026–05–16.

Ocean Networks Canada Society. 2025. Tsawwassen - Duke Point Ferry Route Oxygen Sensor Deployed 2025–06–19. Ocean Networks Canada Society. https://doi.org/10.34943/a33cfb0f-1f85-4ec0-9b1c-64ff919abd09. Accessed 2026–05–16.

Ocean Networks Canada Society. 2025. Tsawwassen - Duke Point Ferry Route Oxygen Sensor Deployed 2025–10–15. Ocean Networks Canada Society. https://doi.org/10.34943/eb0924b9-ff2d-4864-a256-c9b3e424ac8f. Accessed 2026–05–16.

Ocean Networks Canada Society. 2025. Tsawwassen - Duke Point Ferry Route Pump and Valve Control System Deployed 2025–06–19. Ocean Networks Canada Society. https://doi.org/10.34943/e4a9840b-5664-46be-8c54-83902ce08c8f. Accessed 2026–05–16.

Ocean Networks Canada Society. 2025. Tsawwassen - Duke Point Ferry Route Pump and Valve Control System Deployed 2025–10–15. Ocean Networks Canada Society. https://doi.org/10.34943/0f31f356-ad5c-49cf-b886-5c56832d8b4a. Accessed 2026–05–16.

Ocean Networks Canada Society. 2025. Tsawwassen - Duke Point Ferry Route Temperature Sensor Deployed 2025–06–19. Ocean Networks Canada Society. https://doi.org/10.34943/5e0fa038-8c38-4534-ab72-3db104d70b98. Accessed 2026–05–16.

Ocean Networks Canada Society. 2025. Tsawwassen - Duke Point Ferry Route Temperature Sensor Deployed 2025–10–15. Ocean Networks Canada Society. https://doi.org/10.34943/90e3051b-4e9f-46d1-a826-717e2e5ef921. Accessed 2026–05–16.

Ocean Networks Canada Society. 2025. Tsawwassen - Duke Point Ferry Route Thermosalinograph Deployed 2025–06–19. Ocean Networks Canada Society. https://doi.org/10.34943/cfc04eea-bef8-42e4-acea-bbd003f95f0d. Accessed 2026–05–16.

Ocean Networks Canada Society. 2025. Tsawwassen - Duke Point Ferry Route Thermosalinograph Deployed 2025–10–15. Ocean Networks Canada Society. https://doi.org/10.34943/df8bc74a-922f-4321-88fc-c96e743c79f4. Accessed 2026–05–16.

Ocean Networks Canada Society. 2025. Tsawwassen - Duke Point Ferry Route Thermosalinograph Deployed 2025–12–23. Ocean Networks Canada Society. https://doi.org/10.34943/60aba3d2-7ae8-43b8-a1fa-46096e52b59b. Accessed 2026–05–16.

Ocean Networks Canada Society. 2025. Tsawwassen - Swartz Bay Ferry Route Thermosalinograph Deployed 2025–10–10. Ocean Networks Canada Society. https://doi.org/10.34943/9fb1ecdb-2b07-42a3-b4a0-5f423b5db196. Accessed 2026–05–16.

## Data Availability

ZenodoONC-BCF Gridded Sea Surface Ferry Data 2001-2025 (Reference data).
